# The role of ACC deaminase producing bacteria in improving sweet corn (*Zea mays* L. var saccharata) productivity under limited availability of irrigation water

**DOI:** 10.1038/s41598-020-77305-6

**Published:** 2020-11-23

**Authors:** Tayebeh Zarei, Ali Moradi, Seyed Abdolreza Kazemeini, Abdolreza Akhgar, Ashfaq Ahmad Rahi

**Affiliations:** 1 Department of Agronomy and Plant Breeding, Yasouj University, Yasouj, Iran; 2 Department of Plant Production and Genetics, Shiraz University, Shiraz, Iran; 3 Department of Soil Science, Vali-e-Asr University of Rafsanjan, Rafsanjan, Iran; 4 Pesticide Quality Control Laboratory, Multan, Punjab 60000 Pakistan

**Keywords:** Plant physiology, Plant stress responses, Plant symbiosis

## Abstract

Accumulation of stress ethylene in plants due to osmotic stress is a major challenge for the achievement of optimum sweet corn crop yield with limited availability of irrigation water. A significant increase in earth’s temperature is also making the conditions more crucial regarding the availability of ample quantity of irrigation water for crops production. Plant growth promoting rhizobacteria (PGPR) can play an imperative role in this regard. Inoculation of rhizobacteria can provide resistance and adaptability to crops against osmotic stress. In addition, these rhizobacteria also have potential to solve future food security issues. That's why the current study was planned to examine the efficacious functioning of *Pseudomonas fluorescens* strains on yields and physiological characteristics of sweet corn (*Zea mays* L. var saccharata) under different levels of irrigation. Three irrigation levels i.e., 100% (I_100_ no stress), 80% (I_80_), and 60% (I_60_) were used during sweet corn cultivation. However, there were four rhizobacteria strains i.e., *P. fluorescens* P_1_, *P. fluorescens* P_3_, *P. fluorescens* P_8_, *P. fluorescens* P_14_ which were used in the experiment. The results showed that severe water stress (60% of plant water requirement) decreased chlorophyll *a*, chlorophyll *b*, and total chlorophyll contents, *Fv/Fm* ratio and nutrients uptake. A significant increase in F_0_, F_m_, proline, total soluble sugars, catalase (CAT) and peroxidase (POX) activity led to less ear yield and canned seed yield. Combination of four strains significantly increased the yield traits of sweet corn i.e., ear and (44%) and canned seed yield (27%) over control. The highest promoting effect was observed in the combination of four strains treatment and followed by P_1_ strain in reducing the harmful effects of drought stress and improving sweet corn productivity. However, P_14_ gave minimum improvement in growth and yield indices under limited availability of water. In conclusion, combination of four strains inoculation is an efficacious approach for the achievement of better yield of sweet corn under osmotic stress.

## Introduction

Drought is the most important environmental stresses limiting the productivity of crop plants around the world, especially in arid and semi-arid regions^1^. Water stress causes economic damages in agriculture, inoculation of plants with useful microorganisms stimulates plant growth and enhances water stress tolerance^2^. Sweet corn (*Zea mays* L. saccharata) is a hybridized variety of corn specifically bred to increase the sugar content. Sweet corn has a high sugar content; mutation in genes and following that genetic change causes the accumulation of sugars and soluble polysaccharides in the endosperm of the seed^3^. Globally, sweet corn plays an important role, both directly and indirectly, in providing the calories, protein, and some of the vitamins and minerals needed by humans^4^. This crop has been cultivated for a different purpose, such as food for humans, forage for feeding, ethanol, or sugar production in an industry^5^. Sweet corn by producing most structural and soluble sugars, has a very high potential as an alternative raw material for ethanol production; also, the production of biofuels from sweet corn stalk syrup shows that in addition to increasing the value of agricultural losses, this plant can play a temporary role in emerging biofuel markets^6^.

Beneficial bacteria have enormous potential to facilitate plant growth and productivity, in several a number of ways. A remarkable eminence on the credit of plant growth-promoting rhizobacteria (PGPR) such as *Pseudomonas*, *Azotobacter*, and *Azospirillum* is their capability to support plants under stressed environments^7^. Among all, some rhizobacteria are capable to produce ACC deaminase that has the potential to decrease the stress generating ethylene in the plants. Recent studies indicated that rhizobacteria having ACC deaminase are helpful to mitigate the adverse effects of stress generating ethylene^8–14^. Enzyme 1-aminocyclopropane-1-carboxylate deaminase (ACCD), can break ACC, (an immediate precursor of ethylene) to ammonia and α-ketobutyrate^15^. A low level of ACC deaminase activity, namely approximately 20 nmol a-ketobutyrate mg^−1^ h^−1^ is sufficient for bacterium to grow on ACC^16^.

Beneficial effects of PGPR have been reported on reducing the harmful effects of water deficiency on crops growth^1,7,17–19^. Khan et al.^20^ reported elevated activities of CAT, POX, APX, and SOD in response to drought stress; however, under drought stress conditions, inoculation of pea plants with a combination of *Bacillus subtilis*, *Bacillus thuringiensis*, and *Bacillus megaterium* decreased the activity of these enzymes and was significantly increased the amount of chlorophyll, protein, starch, proline, and carbohydrates. Their results indicated that bacteria symbiosis alleviates the toxic effect of drought stress via improving the water status of plants. Danish et al.^21^ in the study of plant growth-promoting rhizobacteria *Pseudomonas aeruginosa*, *Enterobacter cloacae*, *Achromobacter xylosoxidans* and *Leclercia adecarboxylata* in maize plants inoculated under water stress conditions, increasing the concentration of N, P and K nutrients were confirmed by reducing ethylene accumulation and improving plant root growth.

Although numerous kinds of literature have studied the effect of PGPR on growth and drought stress tolerance of maize^22^, however, few studies focus on physiological responses and nutrients uptake sweet corn to PGPR, especially *Pseudomonas fluorescens* under stressful conditions. Therefore, the objective of this study was to evaluate the water stress tolerance that induced in sweet corn by four strains of *Pseudomonas fluorescens*: P_1_, P_3_, P_8_, and P_14_ under different irrigation levels. The current study was conducted with hypothesis that combination of four *Pseudomonas fluorescens* strains inoculation may be more effective approach over sole inoculation for mitigation of adverse effects of limited availability of in sweet corn.

## Materials and methods

### Preparation and inoculation of bacterial strains

Four strains of *P. fluorescens* (P_1 =_ MT949838, P_3_ = MT949840, P_8_ = MT949845, and P_14_ = MT949851) were provided from a bacterial collection of Vali-e-Asr University of Rafsanjan. For analysis of ACC deaminase and auxin synthesis standard protocols were used^23^ (Table 1). Before inoculating *P. fluorescens* strains, the seeds were sterilized for 20 min in 1.5% NaOCl for reducing surface contamination and rinsed with sterile water. The strains were cultured using a test tube (60 ml) on nutrient agar (0.5) and incubated at 27 °C for 48 h. Afterwards, after the transfer of bacteria to the liquid culture medium (TSB), were laid in a shaker incubator at 28 °C for 48 h, and then were centrifuged at about 7000 rpm for 5 min until bacteria precipitate. The optical density of each suspension was determined according to an absorbance at 600 nm of 0.5 in distilled water, with an estimated bacterial cell density 108 CFU ml^−1^^24^. The seeds were placed in the suspensions for 24 h for inoculation with different strains of bacteria.

**Table 1:** Characterization of the *Pseudomonas fluorescens* strains.

Strains	Accession number	Synthesized auxin (µg ml^−1^)	Activity of ACC deaminase enzyme (µmoles mg^−1^ h^−1^)	Mineral phosphate solution (ppm)	Production of siderophores^a^
*P. fluorescens* P_1_	MT949838	7.00 ± 0.02a	3.00 ± 0.012a	538 ± 12a	2.50 ± 0.003a
*P. fluorescens* P_3_	MT949840	6.34 ± 0.07b	2.90 ± 0.030b	484 ± 21c	1.70 ± 0.002c
*P. fluorescens* P_8_	MT949845	6.38 ± 0.12b	2.99 ± 0.080b	507 ± 4b	1.97 ± 0.005b
*P. fluorescens* P_14_	MT949851	5.90 ± 0.05c	2.88 ± 0.040b	405 ± 17d	1.65 ± 0.003d

^a^Halo diameter to colony diameter after 5 days incubation. Values are means of 3 replicates ± standard error compared by LSD at p ≤ 0.05.

Values followed by the same letter (a–d) within the same column are not significantly different at *P* = 0.05.

### Measurement of soil properties

Soil sampling was done according to the zig-zag pattern from depth 0–30 cm sub-samples mixed within the bucket and then 1 kg of soil sample placed in a clean plastic bag and transferred to the laboratory. Soil samples were passed through a 2-mm sieve after being exposed to the laboratory at room temperature for 1 day. The samples were analyzed for soil texture (hydrometric)^25^ and N, P, and K^26^.

### Plant material and growth conditions

**Figure 1:**
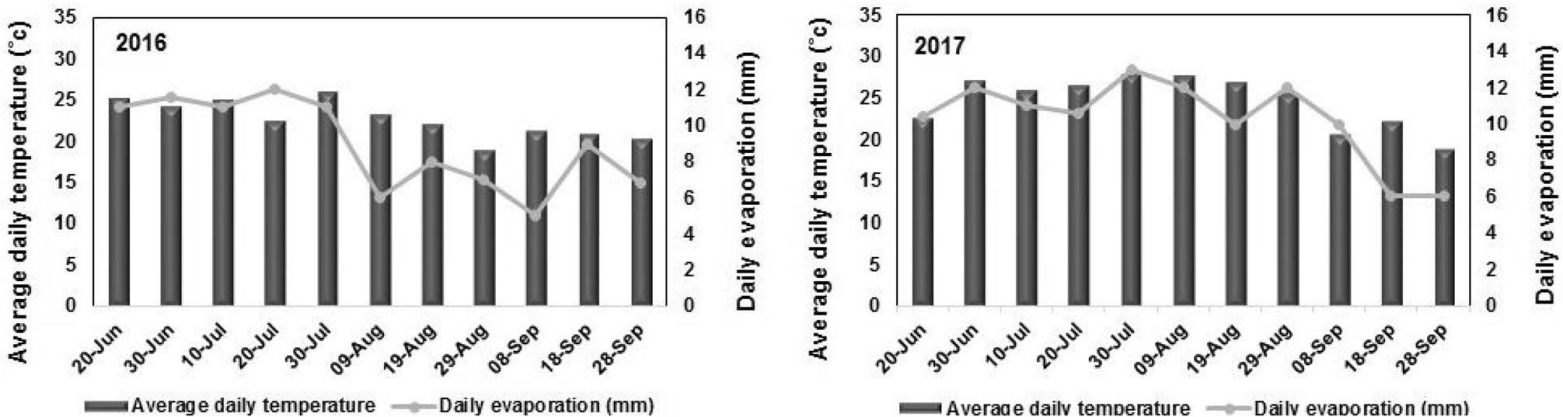
Average *of meteorological data during* the two *growing season* of sweet corn.

This research was conducted in Marvdasht, Fars Province, Iran during 2016 and 2017 growing seasons with semi-arid climate and location coordinates (29° 56′ N, 52° 47′ E). The previous cultivated crop was corn. This region receives a mean annual temperature of 28.5 °C with a mean annual rainfall amount of 365 mm. The soils in Marvdasht are clay-loam classified as (Fine loamy, Carbonatic, Termic, Typic Calcixerpts)^27^. Details of the measured soil properties (0–40 cm depth) before the experiment are provided in Table 2. The field experiment had a completely randomized block design with three replications. The main factor included watering regimes at three levels: 100% (I_100_ no stress), 80% (I_80_), and 60% (I_60_) of plant water requirement) and the subfactor included bacteria inoculation with four strains of *Pseudomonas fluorescens* including *P. fluorescens* P_1_ (P_1_), *P. fluorescens* P_3_ (P_3_), *P. fluorescens* P_8_ (P_8_), *P. fluorescens* P_14_ (P_14_), a combination of four strains (4 Strains) and control). The rhizobacteria were isolated from corn rhizosphere and all strains were drought tolerant (able to grow at − 0.90 Mpa). The Average daily temperature along with daily evaporation is made in Fig. 1. There was no precipitation during growing seasons. Inoculated sweet corn seeds (Chase hybrid) were sown in plots each 3 × 3 m^2^ on 22 June in both years, which rows and plant spacing were 75 and 20 cm, respectively (with 66,000 plants ha^−1^). Based on soil test (Table 2), the broadcast fertilization consisted of 110 kg P ha^−1^ as triple superphosphate and 80 kg K ha^−1^ as potassium sulfate at sowing time and 200 kg N ha^−1^as urea ($${\raise0.7ex\hbox{$2$} \!\mathord{\left/ {\vphantom {2 3}}\right.\kern-\nulldelimiterspace} \!\lower0.7ex\hbox{$3$}}$$ at sowing and the other $${\raise0.7ex\hbox{$1$} \!\mathord{\left/ {\vphantom {1 3}}\right.\kern-\nulldelimiterspace} \!\lower0.7ex\hbox{$3$}}$$ as topdressing at tasseling)^22^.

**Table 2:** Some soil properties of the experimental field before beginning the study (0–30 cm).

Year	Texture	pH	EC (dS m^−1^)	N (%)	P (mg kg^−1^)	K (mg kg^−1^)
2016	Clay loam	7.6	1.41	0.6	13.45	279.0
2017		7.5	1.0	0.7	14.3	297.0

### Methods of applying irrigation treatments

Irrigation was done using a tape system and was similar for all treatments up to complete seedling establishment. Irrigation treatments were applied from 5 leaf stage until the end of experiments. The water requirement of plants was determined based on evapotranspiration using the Penman–Monteith by Eq. (1)^28^.

1 $${\mathrm{ET}}_{0}=\frac{0.408 \Delta \left({R}_{n}-G\right)+\gamma \left[2215/\left(T+273\right)\right]{U}_{2}\left({e}_{s}-{e}_{a}\right)}{\Delta + \gamma \left(1+0.836 {U}_{2}\right)}$$

where ET0 is reference evapotranspiration (mm day^−1^), R_n_ is net radiation at the crop surface (MJ m^−2^ day^−1^), G is soil heat flux density (MJ m^−2^ day^−1^), T mean daily air temperature at 2 m height (°C), U2 wind speed at 2 m height (m s^−1^), e_s_ saturation vapour pressure (kPa), e_a_ actual vapour pressure (kPa), e_s_-e_a_ saturation vapour pressure deficit (kPa), ∆ slope vapour pressure curve (kPa °C^−1^), γ psychrometric constant (kPa °C^−1^). Daily weather information, consisting of a minimum and maximum temperature, precipitations, relative humidity, and sunny h were obtained from Koshkak Meteorological Station (30° 04′ N, 52° 35′ E). Daily water requirement of plants was calculated as follows:

2 $$ {\text{ET}}_{{\text{c}}} = {\text{K}}_{{\text{C}}} \times {\text{ET}}_{0} $$

In this equation, ET_c_, K_C_ and E_T0_ are evapotranspiration of plants (mm day^−1^), crop coefficient, and reference evapotranspiration (mm day^−1^), respectively. According to the FAO report^28^, K_C_ for sweet corn is 1.15 at the middle stage and is 1.05 at maturity. Irrigation depth also was calculated as follows:

3 $$ D = \sum\limits_{i = 1}^{n} {(ET_{Ci} } ) $$

In this equation, D is irrigation depth and ET_ci_ is water requirement. The water volume of each plot was controlled by a water counter.

### Sampling of plants and measurement of traits

For plant sampling, at the milk grain stage, the three last development leaf were randomly selected in each sub-plot. The samples were immediately frozen in liquid nitrogen and stored at − 80 °C for future extraction. Samples Frozen were ground to a fine powder in liquid nitrogen using a pestle and mortar. The concentration of pigments consisted of chlorophyll a & b and total chlorophyll were measured using the spectrophotometer (Shimadzu, JapanUV-3100) at three absorbances (470 nm, 646 nm, and 663 nm) based on Lichtenthaler and Wellburn^29^. Chlorophyll fluorescence parameters (F_0_, F_v_, F_m_, *F*_*v*_/*F*_*m*_) were also read at the milk grain stage by using the fluorimeter (OSI-FL). The content of the free proline was determined according to the acid-ninhydrin method^30^ with minor modification. The determination of soluble sugars was done using Dubois et al.^31^. Catalase^32^ and peroxidase activity^33^ were measured at 240 and 470 nm, respectively, by using the spectrophotometer (Shimadzu, JapanUV-3100).

N content was measured based on the Kjeldahl method^34^, phosphorus by calorimetry method (yellow colour of vanadium molybdate) by using the spectrophotometer (Shimadzu, JapanUV-3100) and potassium by the flame photometer. Micronutrients were also measured by using the dry ash method and then were dissolving in two-normal hydrochloric acid and by using the atomic absorption device (Hitachi ZCAST 2300)^35^.

**Figure 2:**
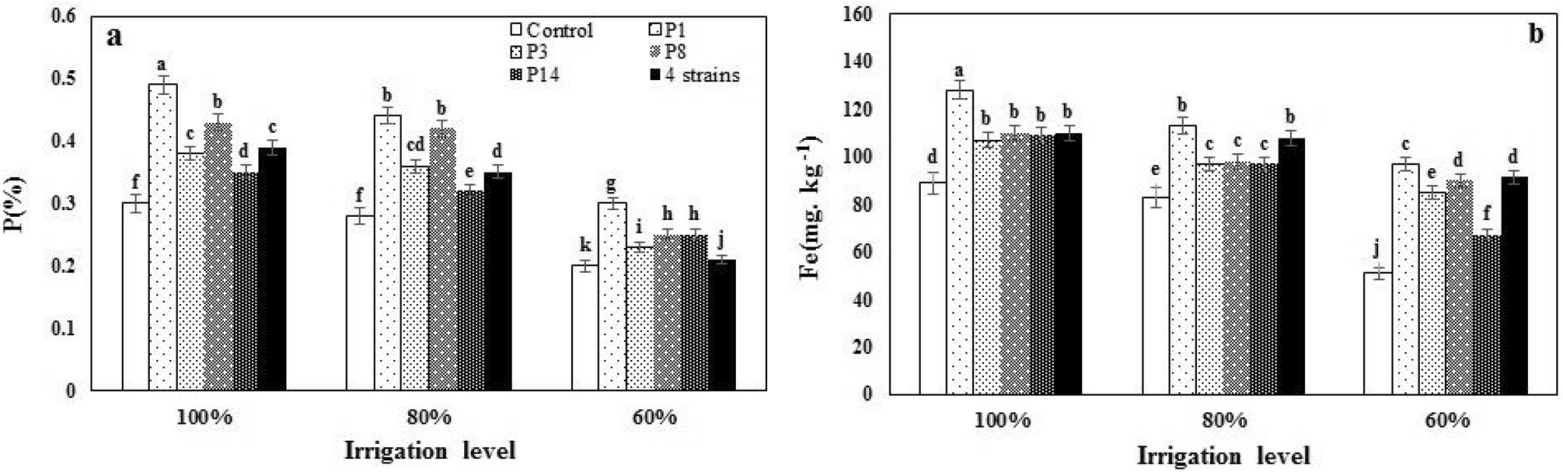
The effect of inoculation with different strains of *Pseudomonas fluorescens* (P_1_, P_3_, P_8_, P_14_ and 4 strains: combination of four strains) under three irrigation levels on P and Fe content of sweet corn leaf. Different letters mean ± standard error bars showed significant difference (LSD at p ≤ 0.05).

At the end of the experiment (i.e., the milky stage of the seeds), 3 m^2^ plants of each plot were harvested manually and ear yield and canned grain yield were determined (Fig. 1 and 2 supplementary file).

### Statistical analysis

Data analysis was performed using SAS software v.9.1 (SAS Institute, Cary, NC, USA). Means of three replicates were compared using the LSD at a P-value of 5%^36^.

## Results

### Macro and micronutrient accumulation in sweet corn leaf

The main effects of irrigation levels (I) and rhizobacteria (B) were significant on nutrients accumulation in sweet corn leaf (except Cu); and the interaction (I × B) was significant only on Fe and P at the probability level of 1 and 5%, respectively (Table 3). Drought significantly reduced leaf nutrients except for K content (Table 5). I_60_ compared to I_100_, resulted in a decrease of 36.0, 32.0, and 39.0% of N, Zn and Mn accumulation of leaf, respectively, and K was increased by 21.98% (Table 5). The use of *Pseudomonas* strains increased the accumulation of leaf nutrients, and a combination of four strains treatment had the most effect and increased content of N, K, Zn, and Mn leaf by 46.04, 43.0, 34.50 and 24.0%, respectively than control (Table 5). I_60_ reduced the P and Fe content of the leaf and compared it to the I_100_, reduced the P and Fe content by 37.0 and 24.5%, respectively (Fig. 2a,b). Bacterial inoculation increased the P and Fe content of the leaf so that the highest and the lowest content of both nutrients were obtained from the P_1_ and control, respectively, at all irrigation levels (Fig. 2a,b).

**Table 3:** Variance analysis of the effects of irrigation levels and bacterial inoculation on nutrients content of sweet corn leaf.

SOV	DF	Mean of squares						
		N	P	K	Fe	Zn	Cu	Mn
Year (Y)	1	0.27	0.010	0.024	108.00	0.326	23.55	38.88
Rep (R)	2	0.009	0.0034	0.122	0.297	0.002	5.11	6.93
Irrigation (Irr)	2	5.50**	0.061**	3.961*	303.68**	13.64*	53.63	74.80**
Y × Irr	2	0.001	ns0.012	0.015	0.022	0.031	0.021	0.018
Error a	4	0.015	0.015	0.044	0.616	0.008	3.00	9.76
Bacteria (Bac)	5	2.13*	0.059**	7.00**	263.41**	35.82**	41.13	52.94*
Irr × Bac	10	0.036	0.069*	0.684	165.41**	18.94	9.41	15.71
Y × Bac	5	0.002	0.022	0.013	0.121	0.101	0.081	0.98
Y × Irr × Bac	10	0.013	0.011	0.010	0.220	0.099	0.090	0.115
Error	60	0.006	0.008	0.005	0.420	0.005	3.13	2.47
C.V.		7.31	4.83	4.86	5.20	4.45	3.95	6.64

*Indicates p ≤ 0.05; ** indicates p ≤ 0.01; C.V. indicates coefficient variance; Rep indicates replication (n = 3); DF indicates degree of freedom; S.O.V indicates source of variance.

### Antioxidant enzymes activity

**Figure 3:**
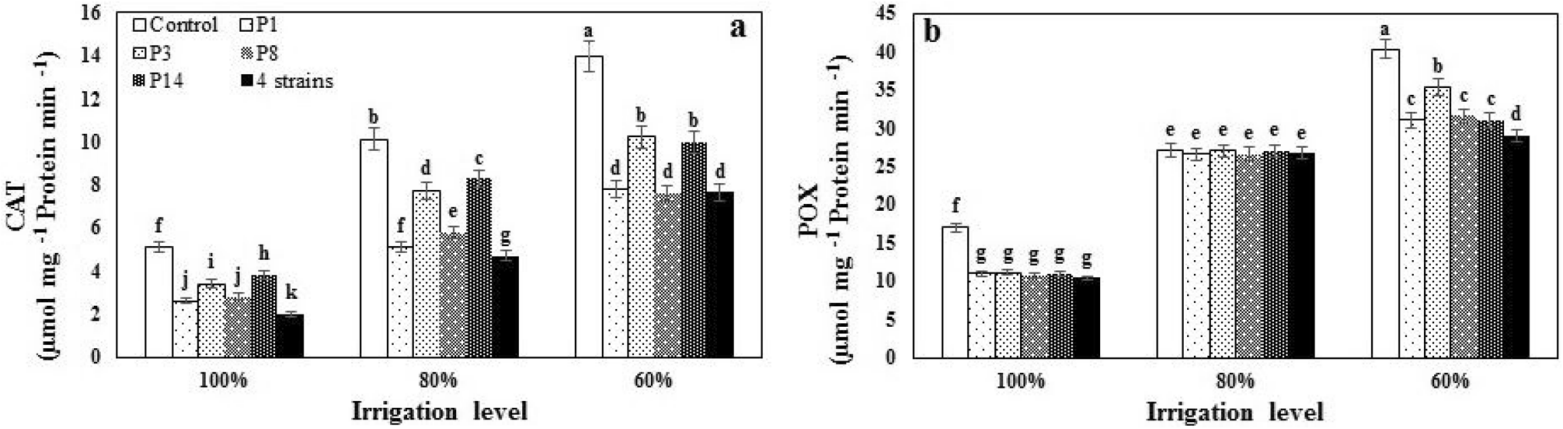
The effect of inoculation with different strains of *Pseudomonas fluorescens* (P_1_, P_3_, P_8_, P_14_ and 4 strains: combination of four strains) under three irrigation levels catalase (CAT) and peroxidase (POX) of sweet a corn leaf. Different letters on mean ± standard error bars showed significant difference (LSD at p ≤ 0.05).

Both the main and interactive effects of I and B were significant in catalase and peroxidase activity (Table 4). Decreasing the volume of irrigation water from the full irrigation to I_80_ and I_60_ were increased catalase and peroxidase activity by 53.0 and 65.50% and 55.0 and 64.00%, respectively (Fig. 3a,b). The use of rhizobacteria moderates the destructive effects of drought stress on the plant and reduces the catalase and peroxidase activity in the plant. At all three irrigation levels, the lowest activity of the catalase and peroxidase was obtained from a combination of four strains treatment, and the control level had the highest activity of both enzymes (Fig. 3a,b).

**Table 4:** Variance analysis of the effect of irrigation levels and bacterial inoculation on physiological traits and yields of sweet corn.

SOV	DF	Mean of squares											
		Chl *a*	Chl *b*	Total Chl	F_0_	F _m_	F _v_ /F _m_	FP	TSS	CAT	POX	Ear Y	Canned SY
Year (Y)	1	0.271**	0.002	0.007	465.84	558.53	0.0042	7.12	13.79	1.428	38.88	67,218.80**	15,830.43**
Rep (R)	2	0.015	0.008	0.003	434.01	55.91	0.0081	20.15	50.01	0.007	0.074	9782.67	2011.03
Irrigation (Irr)	2	0.658**	0.106**	0.670**	2644.72**	576.95**	0.0135**	465.64**	734.54**	532.003**	178.12*	84,745.03**	14,370.63**
Y × Irr	2	0.002	0.004	0.003	32.68	166.17	0.0009	4.53	22.29	0.001	0.011	896.34	1475.09
Error a	4	0.011	0.004	0.003	148.30	122.59	0.0037	19.85	47.12	0.031	0.047	9194.04	1828.22
Bacteria (Bac)	5	0.115**	0.096**	0.036**	78.82**	20.62	0.0009**	105.79**	177.75**	112.31*	240.19**	138,415.30**	72,475.97**
Irr × Bac	10	0.042*	0.019**	0.021**	3.84	410.30**	0.0001	88.55**	246.34*	8.20**	136.25**	35,383.79*	9017.17*
Y × Bac	5	0.011	0.002	0.003	1.45	36.66	0.0001	11.62	24.89	0.002	0.012	9321.52	572.05
Y × Irr × Bac	10	0.008	0.001	0.002	3.16	32.02	0.0001	12.63	36.44	0.002	0.010	3123.87	1370.68
Error	60	0.009	0.002	0.002	61.69	70.06	0.0011	12.04	44.32	0.020	0.023	7280.66	1745.42
C.V.		14.40	13.10	7.64	10.57	3.67	5.13	8.17	11.51	4.17	5.21	7.06	8.56

Chl a: chlorophyll a, Chl b: chlorophyll b, Chl a + b: total chlorophylls, F0, Fm and Fv/Fm: chlorophyll fluorescence parameters, FP: free proline, TSS: total soluble sugars, CAT: Catalase, POX: Peroxidase, Ear Y: ear yield, Canned SY: Canned seed yield.

*Indicates p ≤ 0.05; ** indicates p ≤ 0.01; C.V. indicates coefficient variance; Rep indicates replication (n = 3); DF indicates degree of freedom; S.O.V indicates source of variance.

### Photosynthetic pigments

**Figure 4:**
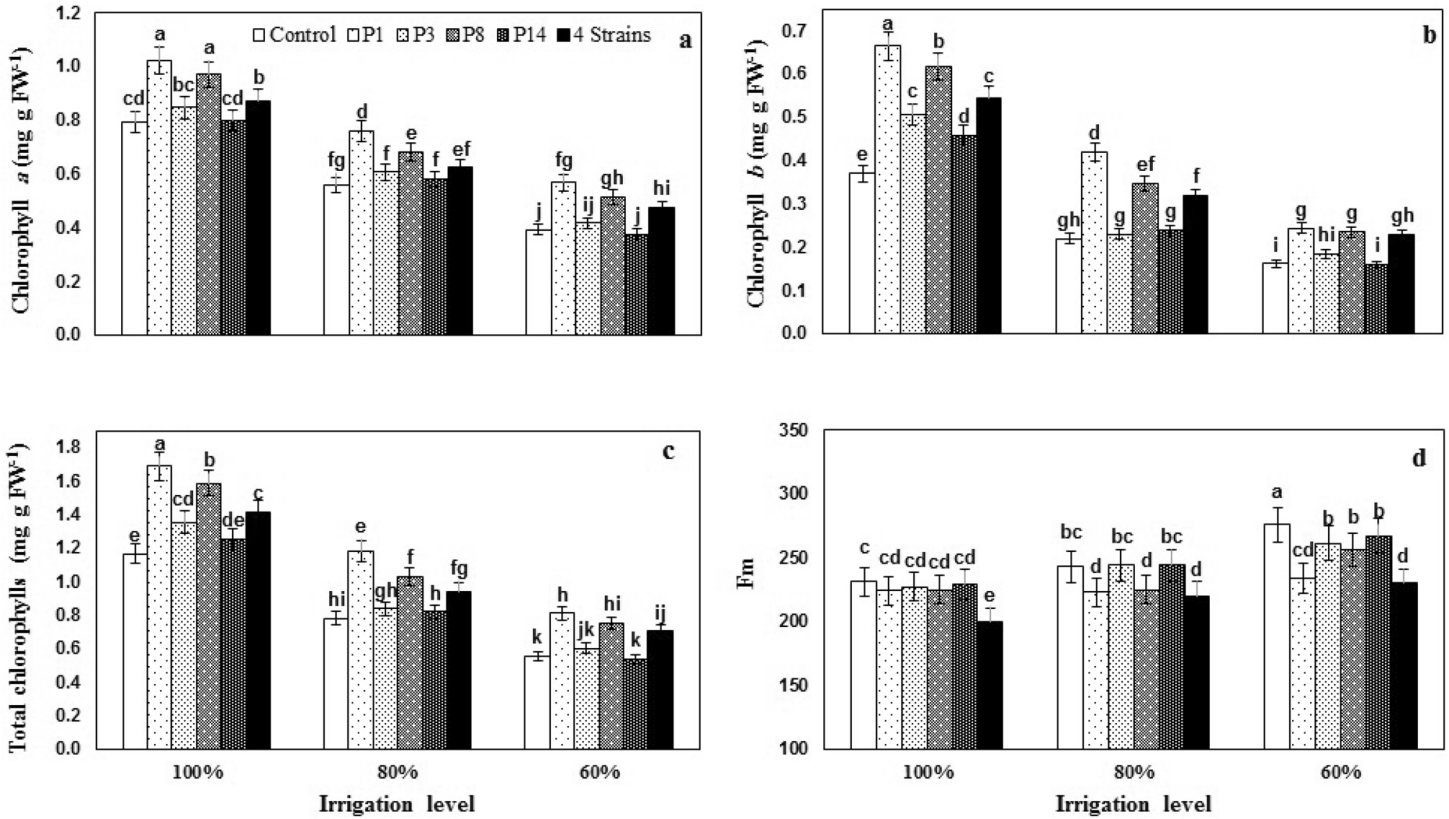
The effect of inoculation with different strains of *Pseudomonas fluorescens* (P_1_, P_3_, P_8_, P_14_ and 4 Strains: combination of four strains) under three irrigation regimes on chlorophyll contents and maximum chlorophyll fluorescence (Fm). Different letters on mean ± standard error bars showed significant difference (LSD at p ≤ 0.05).

Both the individual and interactive effects of I and B were significant on the concentration of chlorophyll *a* (Chl *a*), chlorophyll *b* (Chl *b*), and total chlorophyll, which shown in Table 4. In all irrigation levels, Chl *a* concentration was significantly greater in the seeds inoculated with P_1_ and P_8_ (Fig. 4a), while a combination of all four strain increased Chl *a* concentration only significantly in I_100_ and I_60_ treatments. Inoculation with P_1_ also improved sweet maize water stress tolerance in terms of Chl *a* concentration. So that water stress at I_80_ and I_60_ have associated with 29.4% and 50.4% reductions in Chl *a* at control treatment and 25.7% and 44.6% reductions in P_1_ strain, respectively (Fig. 4a). In no water stress conditions, all strains increased Chl *b* and total chlorophyll concentration; however, in I_80_ and I_60_ conditions only P_1_, P_8_, and combinations of strains had a significant effect (Fig. 4b,c). In all irrigation levels, the highest effects on Chl *b* and total chlorophyll concentrations belonged to P_1_ inoculation. Chl *b* concentration was greater in P_1_ inoculated seeds compared to control by 80, 91, and 51% under I_100_, I_80_, and I_60_ conditions, respectively (Fig. 4b).

### Chlorophyll fluorescence parameters

The main effects of I and B were significant on minimum fluorescence (F_0_) and maximal photochemical efficacy of photosynthetic system (*F*_*v*_*/F*_*m*_) II and also interaction (I × B) was significant on maximum fluorescence (F_m_) of sweet corn leaf (Table 4). Among irrigation levels, the I_60_ level had more F_0_ and increased by 18.0% compared to the I_100_. Among the different strains of *Pseudomonas fluorescens*, the highest and lowest F_0_ was observed by 77.40 and 72.84% in control and combination of four strains treatment, respectively, and the control level was not statistically significant with P_14_ strain (Table 5). Water stress at I_60_ led to a significant increase in F_m_ of sweet corn leaf and F_m_ at this level was 12.35% higher than full irrigation; a combination of four strains treatment resulted in a decrease in F_m_ at all irrigation levels (Fig. 4d).

**Table 5:** Mean comparisons of main effects irrigation regime and bacteria inoculation on chlorophyll fluorescence parameters and nutrients content of sweet corn leaf.

	N (%)	K (mg kg^−1^)	Zn (mg kg^−1^)	Mn (mg kg^−1^)	F_0_	F_v_/F_m_
**Irrigation regime**						
100%	2.17^a^	2.98^b^	36.72^a^	69.19^a^	71.43^b^	0.680^a^
80%	1.73^b^	3.00^b^	32.58^b^	68.75^a^	69.53^b^	0.681^a^
60%	1.39^c^	3.82^a^	24.85^c^	42.27^b^	87.20^a^	0.648^b^
**Bacteria inoculation**						
No bacteria	1.13^b^	2.15^c^	23.27^d^	50.33^d^	77.40^a^	0.668^c^
P_1_	1.94^a^	3.60^b^	33.65^c^	60.00^b^	73.82^c^	0.681^a^
P_3_	1.90^a^	3.62^b^	31.10^b^	55.29^c^	75.72^b^	0.675^b^
P_8_	1.94^a^	3.57^b^	35.00^a^	61.32^b^	74.68^c^	0.679^a^
P_14_	2.00^a^	3.80^a^	35.48^a^	54.00^c^	77.12^a^	0.670^bc^
4-strains	2.10^a^	3.81^a^	35.50^a^	66.28^a^	72.84^d^	0.685^a^

P_1_, P_3_, P_8_, P_14_: four strains of *P. fluorescens*, 4 strains: combination of four strains.

The same letters on each comparison have no significant different (LSD at p ≤ 0.05).

Fv indicates variable fluorescence; Fm indicates maximum fluorescence.

The *F*_*v*_*/F*_*m*_ ratio is an indicator of the photochemical quantum efficiency measurement of photosystem II, which provides a quick and useful way to assess drought stress. At all irrigation levels, the lowest *F*_*v*_*/F*_*m*_ (0.648) was observed at I_60_, and there was a statistically significant difference with other irrigation levels (Table 5). Among *Pseudomonas fluorescens* strains, the highest *F*_*v*_*/F*_*m*_ (0.685) was obtained from the combination of four strains treatment, which was significantly different from strains P_3_, P_14_, and control. However, it was not statistically significant with P_1_ and P_8_ strains (Table 5).

### Leaf proline and total soluble sugars contents

**Figure 5:**
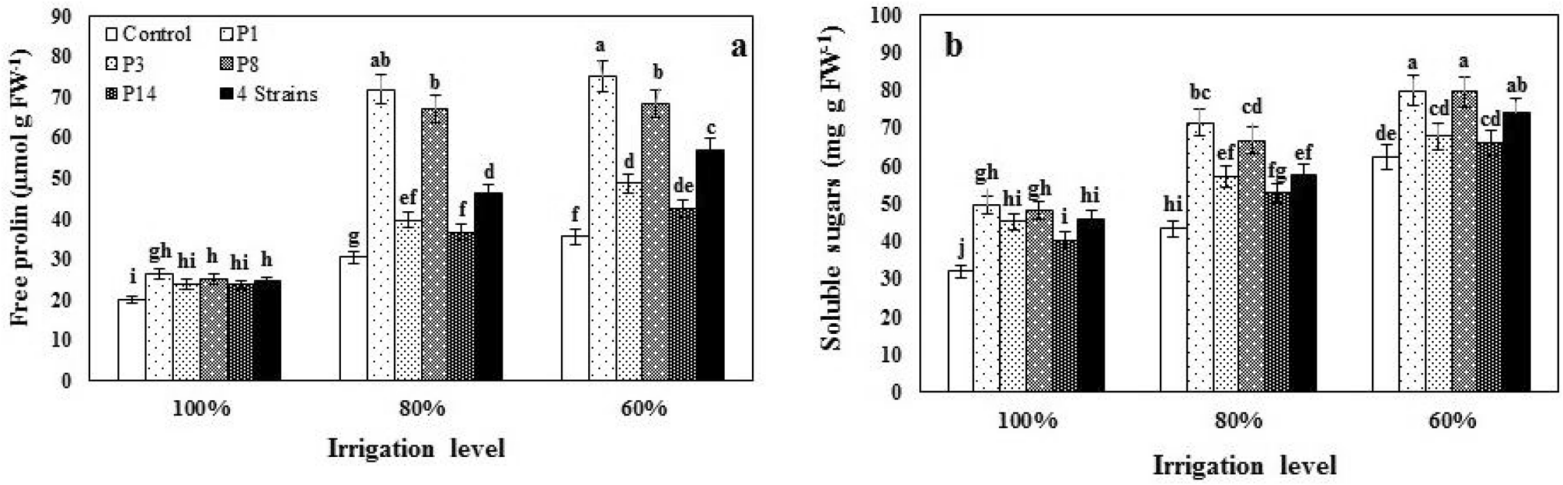
The effect of inoculation with different strains of *Pseudomonas fluorescens* (P_1_, P_3_, P_8_, P_14_ and 4 Strains: combination of four strains) under three irrigation levels on free proline and total soluble sugars. Different letters on mean ± standard error bars showed significant difference (LSD at p ≤ 0.05).

Based on ANOVA results (Table 4), free proline (FP) and total soluble sugars (TTS) of leaves were significantly affected by irrigation regimes, bacteria, and their interaction. Inoculation with P_1_ and P_8_ under no stress conditions and with all strains under both water stress conditions enhanced FP (Fig. 5a). Under the I_60_ water regime, P_1_ as the most effective strain had a greater effect, so that this strain enhanced FP compared with control by 32.0, 136, and 111% under I_100_, I_80_, and I_60_ conditions, respectively. Total soluble sugars were significantly enhanced in response to inoculation with all strains under I_100_ and I_80_ and with P_1_ and P_8_ under I_60_ (Fig. 5b). Inoculated sweet corn with P_1_ had more TSS compared to with control under I_100_, I_80_, and I_60_ conditions by 54, 64, and 28%, respectively.

### Ear and canned seed yield

**Figure 6:**
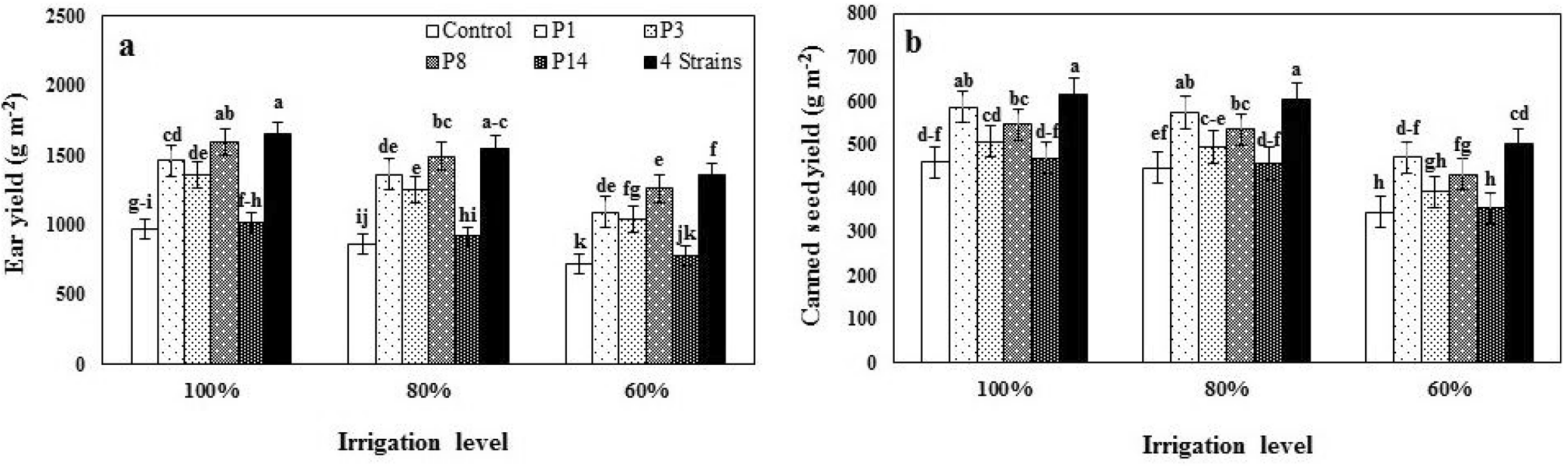
The effect of inoculation with different strains of *Pseudomonas fluorescens* (P_1_, P_3_, P_8_, P_14_ and 4 Strains: combination of four strains) under three irrigation levels on ear yield (Ear Y) and canned seed yield (Canned SY). Different letters on mean ± standard error bars showed significant difference (LSD at p ≤ 0.05).

Both the main and interactive effects of I and B were significant in-ear yield and canned seed yield (Table 4). Reducing irrigation volume to I_60_ was associated with 22.20 and 21.70% reduction in ear yield and canned seed yield, respectively. At all three irrigation levels, inoculation of sweet corn seed with *P. fluorescens* strains improved ear yield and canned seed yield, and the highest ear yield (1651.6 g m^−2^) and canned seed yield (616.1 g m^−2^) were obtained in inoculated plants with a combination of four strains treatment in I_100_ conditions (Fig. 6a,b), that reduced adverse effect of water stress, so that the loss of ear yield at I_60_ were 25.0 and 17.5% in control and combination of four strains level, respectively, and also in canned seed yield from 25% in control reached to 21.0% in 4 Strains level.

## Discussion

Nutrients absorption and transmission mechanisms in plants such as mass flow, dispersion, and osmosis dependence on soil moisture and water reduction in soil cause changes in nutrients absorption^37^. Conformist this fact, present research showed significant nutrients reduction except for potassium at low irrigation I_60_. Nitrogen is one of the most important nutrients in chlorophyll, protoplasm, amino acids, proteins, nucleic acids, and many enzymes. More plant growth indicates more nitrogen absorption. Low irrigation especially at tassel emersion stage is very impressive on nitrogen absorption because of the maximum amount of the nitrogen absorption at this stage and is a critical stage for the crop^38^. It seems that the combination of four strains treatment causes an increase in nitrogen amount of plant shoot followed by improving root growth traits causing more water and nutrients from the soil as a result of auxin production, ACC deaminase productivity, and ethylene production reduction.

Phosphorus is an immobile element in soils. The absorbable phosphorus amount in the rhizosphere is very low. The reason for the fact is mineral phosphate firm ionic bond with soil colloid and fixation as Ferro phosphate or aluminium phosphate which causes phosphorus immobilization in both forms and water stress also reduces this nutrient transmission^39^. Phosphorus solubility and availability reduction, decrease in transpiration, and root system growth and development under deficit soil moisture probably resulted in lower phosphorus amount at I_60_ compare with I_80_ and I_100_ in the present research. Results showed that *P. fluorescens* strains inoculation in all irrigation levels caused more leaf phosphorus and among strains, P_1_ had a higher effect that can be related to the high strength of this strain in mineral phosphate solubilizing (538.0 mg L^−1^) by mineral and organic acids production.

Leaf potassium rising under water deficit can because of its role in cell turgor maintenance^40^. Potassium active absorption is responsible for an increase in its absorbance under water restriction, actually the plant increase potassium density in a shoot with energy consumption unlike the diffusion process for an increase in tolerance against water stress^41^. In the present research, the most potassium amount of leaf was observed at the combination of four strains treatment; it seems that at this level mineral and organic acids production causes more potassium solubility in soil solution by this element ease from biotite and tricalcium phosphate minerals^42^. The strains also impress root growth by IAA hormone production and ACC deaminase activity and finally cause an increase in root absorption and higher potassium amount.

Iron (Fe) is one of the necessary micronutrients for plant growth. Despite there are lots of this element in soil, it is impressed of some soil properties and its mobilization is reduced severely in dry and alkaline soils, therefore will be unavailable for plants. PGPR inoculation under low irrigation caused more Fe amount in the shoot. PGPRs exude chelators with low molecular weight (500–2000 dalton) called siderophore which trends to absorb iron^43^. These materials are exuded from the microbial cell for against Fe absorbable form deficit (< 20 µM) and make this element to the soluble chelate form, therefore iron mobilization in the rhizosphere is increased and absorbed by plant root system with special mechanisms. After that roots can absorb iron from Fe-siderophore that this process can conduct by different mechanisms such as chelate destruction follow by Fe loose, direct absorption from Fe-siderophore or ligand conversion reaction^44^. In this research, the most amount of Fe achieved from P_1_ strain and it can because of the high ability of this strain to produce siderophore (2.5 halo diameter to colony diameter) compare with other strains and mentioned strain caused more Fe absorbability by the iron chelate formation and more shoot Fe amount.

Zinc plays an important role in protein and carbohydrates synthesis and effects on saccharides, nucleic acids, and lipids metabolism^45^. Calcareous soils (high pH) under low irrigation causes a severe reduction in micronutrients especially zinc solubility and also being the high amount of bicarbonate in water, especially under low irrigation, results from more bicarbonate concentration in cellular sap follow by more pH in sap and zinc sediment in vessels and so lower zinc transformation to the rest parts of plants^43^. In the present research, among different strains of *P. fluorescens*, the most amount of leaf zinc achieved from the combination of four strains treatment. It seems that this level of strains can change insoluble zinc forms into soluble forms by organic acids production, increase in proton (H^+^) and chelating compounds making. The main role of these bacteria on zinc solubility is pH reduction up to 5 or less and then more availability of the element. Also, the increasing effect of strains under combination inoculation increases root contact surface with soil and shorter element transform to roots^46^. Zinc and Fe compete for absorption from the root and the combination of four strains level was effective on Fe absorption more than zinc because of the most siderophore production and chelate Fe-siderophore formation.

The main role of Mn in plants is in photosynthesis and oxygen production (Hill reaction). Mn plays an electron receptor transformer role in photosystem II during the primary phase of photosynthesis. Another role of Mn is participation in antioxidant enzymes which restricts free radicals activity so prevents lipids destruction like glycolipids and membrane fatty acids^47^. Iran soils have deficit micronutrients because of being classical, bicarbonate water, and low organic matter, also under low irrigation Mn solubility becomes less and its absorption and transformation into plants will be lower and finally Mn amount in shoot decreases. The combination of four strains level improved plant ability for nutrients absorption like Mn by expanding the host root system and increasing root contact with soil as a result of more lateral root and root hair production. Abbaszadeh-Dehaji et al.^48^ reported that three PGPRs (*Pseudomonas putida* P159, *Pseudomonas flurescens* T17-24 and *Bacillus subtilis* P96) inoculation resulted from a significant increase in sorghum growth and nutrients like Mn, Zn, Cu, and K in plant leaf in compare with control plants. Malekzadeh et al.^49^ showed that Fe, Zn, P and Mn absorption by maize shoot increased significantly in all inoculated treatments with *Bacillus mycoides* and *Micrococcus roseus* in compare with control.

Under water stress, plants have more antioxidant enzyme activity. Under that situation oxygen active species production increases because of stomata closing and CO_2_ absorbance and fixation deficit, as a result antioxidant enzymes activity gets higher to eliminate oxygen active species^50^. *P. fluorescens* strains inoculation caused a reduction in catalase and peroxidase activities in leaf. Both enzymes activities reduction under strains combined inoculation can be explained by this fact that the combination of four strains level by stress tolerance related genes induction causes transpiration decrease by leaves roll and stomata regulation. Also, this level prevents high enzyme activity by cell water maintenance, photochemical efficiency improvement of photosystem II, electron final receptor reduction preventing and active oxygen species decrease^51^. Under water stress condition, PGPRs (*Pseudomonas jessenii* R62, *Pseudomonas synxantha* R81, *Arthrobacter nitroguajacolicus* YB3 and *Arthrobacter nitroguajacolicus* YB5) using caused plant growth promotion and reduction in superoxide dismutase, catalase, peroxidase, and acrobate peroxidase activity in two species of drought-tolerant rice (Sahbhagi) and drought-sensitive species (IR-64) in comparison with control plants^52^.

The results showed that photosynthetic pigments consisted of chlorophyll *a* and chlorophyll *b* were significantly reduced by water stress, which intensifies stress level and increased its adverse impact. It was also shown that the sensitivity of chlorophyll *b* was the most. The most chlorophyll lost in response to drought stress happens in the mesophyll cells compared to the bundle sheath cells^53^. Reduced chlorophyll concentration mainly is known to be due to reactive oxygen species in oxidative stress^54^, which have been reported in different plants such as sweet corn by Singh and Shinde^1^, sunflower by Manivannan et al.^55^ and mung bean (*Vigna radiata*) by Farooq and Bano^56^. Under water deficit stress conditions, chlorophyll concentration is reduced, which may be because chlorophyll degradation is more than chlorophyll synthesis.

Although bacteria inoculation increased photosynthetic pigments concentration, however, there was a clear cut difference between strains, so that P_1_ and P_8_ strains had the most effect and P_14_ had no significant effect. A combination of four strains had no advantages than individual use of them. The improved concentration of chlorophylls under water deficit stress in *P. fluorescens* inoculated plants might be probably due to greater synthesis of photosynthetic pigments through higher stomatal conductance and increase photosynthetic area or also through enhanced water and nutrients absorption^57^. Strains of *P. fluorescens* especially P_1_ and P_8_ enhanced concentration of Chl *a* and Chl *b*. Inoculation with P_1_ not only enhanced Chl *a* concentration but also improved water-deficit stress tolerance of plants at both water stress levels. This improvement was observed for Chl *b* only in moderate water stress. Prolonged growth cycle and consequently higher green stay and delaying aging might be the main reason for the enhancement of chlorophylls concentrations in inoculated plants^58–60^, which could be due to synthesized auxin as a result of inoculation with *P. fluorescens* strains. In the current research, all evaluated strains had a considerable activity of ACC deaminase (Table 1). In the case of Chl *b* concentration, water deficit stress discomfited the positive role of P_3_ and P_8_ inoculation, so that these two strains had significant on Chl *b* only in no stress conditions.

Chlorophyll florescence has been suggested as a criterion for environmental stress effects on the measurement of plants and their tolerance against stress determination. This factor can show these stresses on how much hurt photosynthetic systems^61^. In one research, water stress caused leaf water content reduction, higher minimum florescence of chlorophyll by electron transforming chain damage in photosystem II following by Quinone (QA) capacity decrease and uncompleted oxidation because of slow electron afflux in photosystem II and finally, this photosystem becomes inactivated^62^. It seems the combination of four strains level resulted in promotion in photosystem II electron transforming chain efficiency by synergistic effects of strains on root growth properties improvement and higher root contact surface and better water and nutrients absorption leading to lower minimum florescence^63^. Stomata closing after low water potential in leaf has a bad effect on CO_2_ entrance and decreases electron reception and transferring capacity, in a result system attains maximum florescence immediately; in response, variable florescence is reduced^64^. It can be concluded that under low irrigation (I_80_ & I_60_), strains combined inoculation treatment causes plant water condition improvement and alleviation in low water effects by root system expanding and more root contact surface with soil, which in response maximum florescence would decrease in comparison with no inoculated treatment.

*Fv/Fm* is an index for photosystem II photochemical quantum efficiency measurement that is a fast and useful way to investigate drought stress^65,66^. The decrease in CO_2_ assimilation because of stomata closing under drought stress causes no consumption for electron transferring chain outputs (ATP and NADPH) and so reduced ferodoxine is increased and it causes more active radicals production, these radicals destroy tilacoid membrane proteins and prevent electron transferring from reception place of photosystem II leads to slower electron transferring speed and lower photosystem II photochemical efficiency^67^. The combination of four strains treatment results in different mechanisms like IAA production which leads to root cell propagation and elongation, using ACC enzyme that applies ACC preform for ethylene production and by hydrolyzing ACC to ammonia and α-chetobutirate causing lower ethylene prevention level and more root growth. Also solubilizing of mineral phosphates and siderophore production and Fe supplement for plant causes more water and nutrients absorption for photosynthesis and because of that electron flowing from photosystem II to photosystem, I would be better and maximum photochemical efficiency of photosystem II will increase^68^.

Increased free proline, as well as total soluble sugars, were observed in stressed plants, and these changes depending on the severity of water deficit stress. One of the most important plant responses to water deficiency is an osmotic adjustment that mentions the decreased osmotic potential of the plants by accumulation solutes like proline and sugars^7^, which shown in the current study. Proline and soluble sugars are acting as osmolytes sustaining cell turgor of leaves, protecting the integrity of the membrane, and preventing the denaturation of proteins^69,70^. The mechanism of bacteria inoculation on proline has not well-cleared yet. Bacteria inoculation significantly enhanced the content of FP and TSS, and this effect was varied between strains and irrigation levels. On average, all strains had positive effects; however, P_1_ and P_8_ were the most effective of those.

Experimental evidence indicated that the activity of ACC deaminase was the key factor in the ability of PGPR to stimulate the elongation of plant roots^15^. The moderate activity of ACC deaminase causes a balance between ACC deaminase activity and root growth^23^. It seems under water stress conditions, plants inoculated invest a large amount of carbon and nitrogen resources into synthesis compatible solutions in the leaves such as proline for maintaining cell turgor by improving root growth and increasing the area of nutrient uptake.

Overall results showed that sweet corn ear and canned seed yields were decreased in moderate and severe water stress levels. Previous studies also reported that water stress has been led to less growth and yield in sweet corn^1,5^. The yield of sweet corn consisted of ear yield and canned seed yield was greater in plants inoculated with *P. fluorescens*. The main mechanisms of *P. fluorescens* to facilitate plant growth and development is the lowering of the ethylene levels by hydrolysis of 1-aminocyclopropane-1-carboxylic acid (ACC), the immediate precursor of ethylene in plants^19^. In this study, water stress at I_60_ has influenced the production of assimilates and ear beginnings by reducing the absorption of water and nutrients, especially N, P and K and leaf area expansion, as a result, has led to a decrease in the number of rows in the ear, the number of seeds per row, the weight of 1000 seeds and finally ear and canned seed yields.

At all levels of irrigation, the combined inoculation of strains has affected root growth characteristics by their high ability to inhibit ethylene hormone and prevent its inhibitory effect on root growth and in terms of water and nutrients, they provide a large area of soil for the plant; and increasing the concentration of photosynthetic pigments and improving the photochemical efficiency of photosystems has led to an increase in assimilates and following that an increase in the number of ears, the number of seeds per ear, the weight of 1,000 seeds, and thus the canned seed yield. It has been well-accepted that root growth, especially under water deficit conditions could lead to more photosynthesis rate and so higher yield^7,18,20,24,71,72^.

## Conclusion

Water scarcity is a limiting factor for agricultural productivity that causes changes in physiological characteristics and nutrients uptake in sweet corn. Inoculation with different strains of *P. fluorescens* improved physiological attributes and increased yield. Combination of four strains treatment through the synergistic effects of strains in the production of ACC deaminase, auxin synthesis, the ability in mineral phosphate solubilizing and the production of siderophore significantly improved the yield traits of sweet corn at different levels of irrigation by reducing the effects of stress ethylene and increasing water absorption and nutrients.

## Supplementary information

Supplementary Information.
