# Supplementary material for: The role of ACC deaminase producing bacteria in improving sweet corn (Zea mays L. var saccharata) productivity under limited availability of irrigation water
Source: Sci Rep. 2020 Nov 23;10:20361. doi: 10.1038/s41598-020-77305-6 (PMC7683742; doi:10.1038/s41598-020-77305-6)
Supplement: Supplementary file 1 — Supplementary Information. [file 41598_2020_77305_MOESM1_ESM.docx]

**The role of ACC deaminase producing bacteria in improving sweet corn (*Zea mays* L. Var saccharata) productivity under limited availability of irrigation water**

Tayebeh Zarei ^a^, Ali Moradi ^a*^, Seyed Abdolreza Kazemeini ^b^, Abdolreza Akhgar ^c^ and Ashfaq Ahmad Rahi^d^

^a^ Department of Agronomy and Plant Breeding, Yasouj University, Yasouj, Iran

^b^ Department of Crop Production and Plant Genetic, Shiraz University, Shiraz, Iran

^c^ Department of Soil Science, Vali-e-Asr University Of Rafsanjan, Rafsanjan, Iran

^d^ Pesticide Quality Control Laboratory, Multan, 60000 Punjab, Pakistan

| 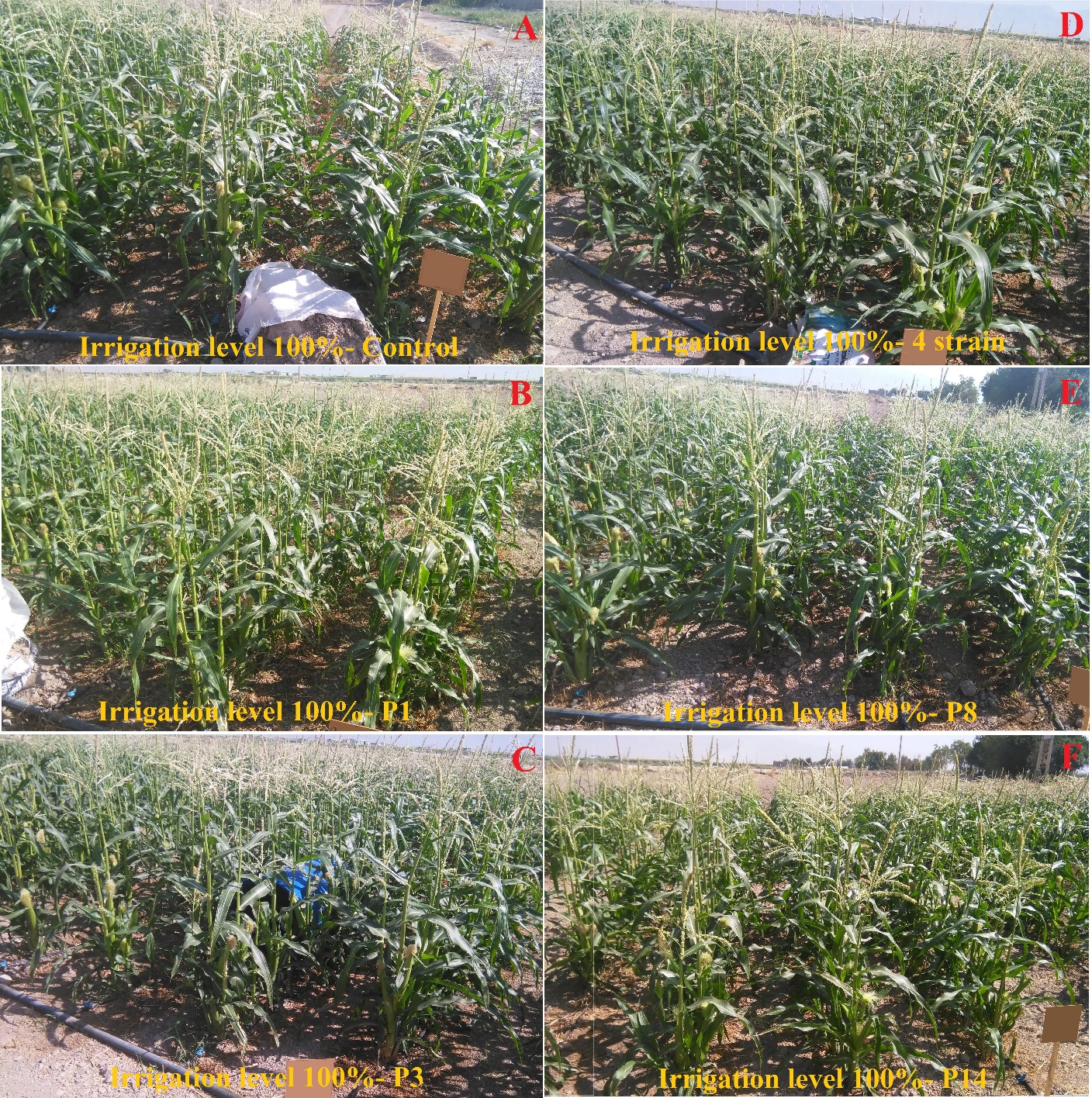 |
| --- |
| **Figure 1.** Effect of irrigation level in different levels of *Pseudomonas fluorescens* (P_1_, P_3_, P_8_, P_14_ and 4strains: combination of four strains). **(A)** Irrigation level 100%- Control; **(B)** Irrigation level 100%- P1; **(C)** Irrigation level 100%- P3; **(D)** Irrigation level 100%- 4Strains; **(E)** Irrigation level 100%- P8; **(F)** Irrigation level 100%- P14.  Captured by 1^st^ author |

| 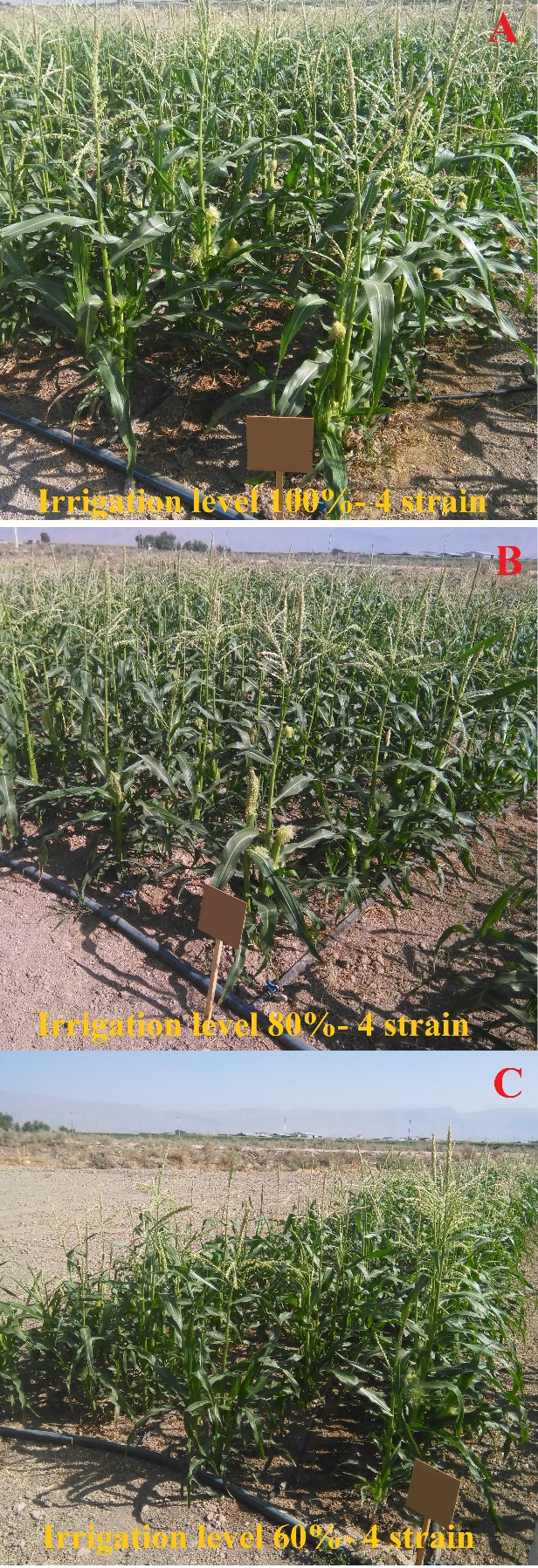 |
| --- |
| **Figure 2.** Effect of three irrigation levels in treatment 4strains (combination of four strains). **(A)** Irrigation level 100%- 4strains; **(B)** Irrigation level 80%- 4strains; **(C)** Irrigation level 60%- 4strains  Captured by 1^st^ author |
